# Basic and Translational Research in Cardiac Repair and Regeneration: *JACC* State-of-the-Art Review

**DOI:** 10.1016/j.jacc.2021.09.019

**Published:** 2021-11-23

**Authors:** Jianyi Zhang, Roberto Bolli, Daniel J. Garry, Eduardo Marbán, Philippe Menasché, Wolfram-Hubertus Zimmermann, Timothy J. Kamp, Joseph C. Wu, Victor J. Dzau

**Affiliations:** a Department of Biomedical Engineering, School of Medicine, School of Engineering, The University of Alabama at Birmingham, Birmingham, Alabama, USA;; b Institute of Molecular Cardiology, University of Louisville, Louisville, Kentucky, USA;; c Department of Medicine, Lillehei Heart Institute, University of Minnesota, Minneapolis, Minnesota, USA;; d Smidt Heart Institute, Cedars-Sinai Medical Center, Los Angeles California, USA;; e Department of Cardiovascular Surgery, Hôpital Européen Georges Pompidou, University of Paris, PARCC, INSERM, F-75015, Paris, France;; f Institute of Pharmacology and Toxicology, University Medical Center Göttingen, and DZHK (German Center for Cardiovascular Research), partner site Göttingen, Göttingen, Germany;; g Department of Medicine, School of Medicine and Public Health, University of Wisconsin-Madison, Madison, Wisconsin, USA;; h Stanford Cardiovascular Institute, Stanford University School of Medicine, Stanford, California, USA;; i Mandel Center for Hypertension Research, Duke Cardiovascular Center, Duke University School of Medicine, Durham, North Carolina, USA.

**Keywords:** heart, heart failure, myocardial infarction, myocyte proliferation, pluripotent stem cell

## Abstract

This paper aims to provide an important update on the recent preclinical and clinical trials using cell therapy strategies and engineered heart tissues for the treatment of postinfarction left ventricular remodeling and heart failure. In addition to the authors’ own works and opinions on the roadblocks of the field, they discuss novel approaches for cardiac remuscularization via the activation of proliferative mechanisms in resident cardiomyocytes or direct reprogramming of somatic cells into cardiomyocytes. This paper’s main mindset is to present current and future strategies in light of their implications for the design of future patient trials with the ultimate objective of facilitating the translation of discoveries in regenerative myocardial therapies to the clinic.

Exactly 20 years ago, progenitor cells, in fact myoblasts from a skeletal muscle, were for the first time injected into a human heart during a concomitant coronary artery bypass grafting in a patient with severe heart failure (HF) (1). This procedure opened an era during which multiple experimental and clinical studies have been performed. The main conclusion is that despite the initial hopes of “regenerating” the failing heart, outcomes have usually been neutral or at best marginally positive regarding clinically relevant end points regardless of the cell type, dosing, and route for delivery, thereby suggesting that when present, efficacy falls far short of remuscularization of the injured heart. Importantly, however, several lessons have been drawn from all of these studies that fuel the current research on cardiac cell therapy. As the field has been the subject of multiple reviews and meta-analyses, the objective of this paper is not to duplicate these publications. Rather, we will first focus on the most recent clinical trials, then address the current trend for using exclusively cell-derived products, and finally provide a glimpse into the future by discussing some of the perspectives offered by generation of endogenous cardiomyocytes (CMs) to achieve the yet unachieved objective of replacing the myocardial scar with integrated, functional cardiac muscle.

## AN OVERVIEW OF THE LATEST CLINICAL TRIALS

Clinical trials of cell-based therapies for heart disease have been recently reviewed extensively in the literature (2,3), and therefore will not be elaborated here. Overall, the adult cell therapy was found to be safe and to have low immunogenicity; there was usually no significant effect on infarct size, LV function, or clinical outcome, but the phase I/II studies were not powered for efficacy. The emerging paradigm is the possibility that exogenous cells may exert most of their beneficial actions via an immune-modulation, as discussed later in the text. This concept is supported by the fact that HF is associated with chronic inflammation, and that these clinical trials have often used cells endowed with anti-inflammatory actions. Clearly, much work remains to be done, and well-designed phase III trials will help determining the efficacy of cells or their products for the treatment of heart disease. Furthermore, although most of the previous clinical trials have used single-dose intervention, going forward, the expectation that such a single administration is sufficient to impart a long-term beneficial effect is probably unrealistic. Another emerging paradigm is thus the interest of using the intravenous route whose lack of invasiveness is compatible with repeat dosing with the premise that sequestration of the cells in lungs, liver, or spleen may not preclude them to exert remote cardiac benefits via systemically-induced anti-inflammatory actions.

### MESENCHYMAL STROMAL CELLS AND CARDIAC C-KIT POSITIVE CELLS FOR TREATMENT OF PATIENTS WITH HF.

Despite misconduct in 1 laboratory, at least 50 studies from 26 independent research groups have established the benefit of c-kit positive cells (CPCs) on left ventricular (LV) function in animal models (eg, mice, rats, pigs, and cats) of ischemic heart disease (3). Contrary to initial expectations, engrafted CPCs do not differentiate into CMs; rather, they act similarly to most other cell types—by stimulating paracrine mechanisms that may reduce inflammation, fibrosis, and apoptosis while promoting angiogenesis. Bone marrow (BM)–derived mesenchymal stromal cells (MSCs) have also been shown to exert beneficial effects in animal models of ischemic heart disease (2), and single-center phase I and II trials in patients with ischemic HF have been encouraging. Like CPCs, MSCs act via the secretion of paracrine factors (2), and the recently completed phase III DREAM-HF trial, which has randomized 565 patients to endoventricular injections of allogeneic MSCs or placebo, should soon provide additional insights into the effects of this therapy. Furthermore, there is preclinical evidence that the benefits of MSCs and CPCs for the treatment of ischemic cardiomyopathy may be additive (2,4), but no clinical trial had examined a combination of different cell types for treatment of HF before the CONCERT-HF (Combination Of meseNchymal and c-kit^+^ Cardiac stEm cells as Regenerative Therapy for Heart Failure) trial.

CONCERT-HF was a phase II, randomized, double-blind, placebo-controlled, multicenter trial by the National Heart, Lung, and Blood Institute Cardiovascular Cell Therapy Research Network that investigated the feasibility, safety, and efficacy of autologous BM-derived MSCs and CPCs for treatment of patients with HF caused by ischemic cardiomyopathy (5). The trial sought to answer the following questions: is combined treatment with autologous MSCs and CPCs feasible and safe in patients with ischemic HF; do MSCs and CPCs, alone or in combination, have beneficial effects on major adverse cardiac events, quality of life, functional capacity, LV function, and/or scar size; is one cell type more effective than the other; and is the combination of MSCs and CPCs superior to MSCs alone or CPCs alone? CONCERT-HF was the following: 1) the first randomized, double-blind, placebo-controlled, multicenter clinical trial to evaluate CPC administration in ischemic HF patients using cell products manufactured under good manufacturing practices standards in a facility accredited by the U.S. Food and Drug Administration; 2) the first multicenter, randomized controlled trial to evaluate the effects of unmodified MSCs for treatment of HF; and 3) the first clinical trial to evaluate a combination of 2 different cell types obtained from different tissue sources in patients with ischemic HF (5).

A total of 125 patients with ischemic HF were randomized to transendocardial injection of both MSCs and CPCs, MSCs alone, CPCs alone, or placebo and followed for 12 months. Autologous MSCs were obtained by BM aspiration, and autologous CPCs by endomyocardial biopsy. At baseline, average left ventricular ejection fraction (LVEF), assessed via cardiac magnetic resonance imaging, was 28.6 ± 6.1%, and average scar size was 19.4 ± 5.8% of the LV; 80% of patients were in New York Heart Association functional class II and 15% were in New York Heart Association functional class III. All participants were on maximally tolerated, guideline-directed therapy. Cell administration was safe: there were no significant differences in adverse event occurrence across the treatment groups. Efficacy outcomes were evaluated by intention-to-treat analysis, and because of the exploratory nature of the study, no adjustments for multiplicity were made. At either 6 or 12 months after treatment, there were no significant differences between treated and placebo groups in LVEF, LV volumes, global circumferential strain, longitudinal strain, sphericity index, scar size, peak VO_2_ (treadmill test), 6-minute walking distance, or levels of N-terminal pro-B-type natriuretic peptide. However, there were significant differences in clinical outcome. The incidence of heart failure–related major adverse cardiac events (HF-MACE) (defined as all-cause death, hospitalization for worsening HF, or HF exacerbation not requiring hospitalization) differed significantly across the 4 groups *(P* = 0.049), with the highest incidence observed in the placebo group (28.1%) and the lowest in patients who received CPCs alone (6.5%; *P* = 0.043 vs placebo). HF-MACE were also reported in 9.1% of patients in the MSCs + CPCs group *(P* = 0.061 vs placebo). The differences in HF-MACE occurrence were driven primarily by hospitalization for HF, which declined from 21.9% of patients in the placebo group to 3.2% in the CPCs alone group *(P* = 0.053 vs placebo) and 6.1% in the MSCs + CPCs group *(P* = 0.082 vs placebo). Compared with the placebo group, the HR was significantly lower in the CPCs alone group (HR: 0.200 [95% CI: 0.043–0.934]; *P* = 0.041) and in the MSCs + CPCs group (HR: 0.256 [95% CI: 0.069–0.934]; *P* = 0.043) (Cox regression analysis). Other cardiovascular clinical events were not significantly different among groups. The Minnesota Living with Heart Failure Questionnaire score at month 6 was also better in the MSCs alone group *(P* = 0.050) and the MSCs + CPCs group *(P* = 0.023) than in the placebo group at month 6, and in the MSCs + CPCs group than in the placebo group at month 12 *(P* = 0.020).

Taken together, the results of CONCERT-HF suggest that in patients with chronic ischemic HF on maximal guideline-driven therapy, a single administration of autologous CPCs or MSCs had measurable beneficial effects over the ensuing 12 months, including a decline in hospitalization for HF (with CPCs) and an improvement in quality of life (with MSCs) (6). The best overall results were obtained by combining MSCs with CPCs, which both reduced HF-MACE incidence (HR: 0.256; *P* = 0.043) and improved quality of life (Minnesota Living with Heart Failure Questionnaire score; *P* = 0.023). Since neither CPCs nor MSCs, alone or in combination, improved LV function or reduced scar size, further research will be needed to elucidate the mechanism(s) underlying the beneficial effects of CPCs and MSCs, such as possible anti-inflammatory/antifibrotic activity and/or improvement in endothelial function. Nevertheless, these findings suggest that CPCs and MSCs have disease-modifying properties in ischemic HF.

### CARDIOSPHERE-DERIVED CELLS FOR TREATMENT OF PATIENTS WITH DUCHENNE MUSCULAR DYSTROPHY.

Cardiosphere-derived cells (CDCs) are a type of heart stromal/progenitor cell that has entered testing in advanced-phase clinical trials. CDCs have potent immunomodulatory, antifibrotic, and regenerative activity in both diseased hearts and skeletal muscle (7), and to date, they have been the subject of >250 peer-reviewed papers from >65 independent laboratories worldwide (PubMed search on April 29, 2021) (7). Allogeneic CDCs have been consistently associated with a favorable safety profile and indications of disease-modifying bioactivity when tested in 7 clinical trials, and the most advanced clinical CDC program targets Duchenne muscular dystrophy (DMD), which affects both the heart and skeletal muscle (8). Two DMD clinical trials, HOPE and HOPE-2 (Halt cardiOmyoPathy progression) (NCT02485938 and NCT03406780, respectively) have been performed with CDCs. In HOPE, patients were treated with a single dose of CDCs via multivessel coronary infusion, and the treatment was associated with improvements in both cardiac scar size and measures of upper-limb performance when compared with treatment via the standard of care (9). Notably, preclinical studies in a mouse DMD model (*mdx* mice, which carry a naturally occurring mutation in the dystrophin gene) suggested that intravenously (IV) delivered CDCs led to profound functional improvements in cardiac and skeletal muscle (10) and that the benefits of repeated CDC doses were cumulative (8). Thus, the phase II randomized double-blind placebo-controlled HOPE-2 trial was designed to evaluate 4 doses of CDCs (or placebo) delivered IV at 3-month intervals in 20 patients with advanced DMD, most of whom were nonambulatory. Phenotyping was conducted via cardiac magnetic resonance imaging and by quantitative assessment of upper-limb performance.

A preliminary presentation of HOPE-2 study results (11) reported that the CDC treatments were safe and, at 1 year, associated with major improvements in a variety of cardiac parameters, including significant differences between the CDC- and placebo-treatment groups in LVEF *(P* = 0.004) and greater reductions in LV chamber dimensions including LV end-systolic volume (Δ = 6.31 mL; *P* = 0.0045), which is often used as a surrogate parameter for LV remodeling in clinical trials. Measures of the MB isozyme of creatine kinase were also markedly lower in CDC-treated than in placebo-treated patients, which suggests that CDC administration protected cardiac muscle from damage over the course of the study. Thus, HOPE-2 was the following: 1) the first clinical trial to evaluate cell therapy delivered via a repeated sequential dosing regimen for any cardiac indication; 2) the first to evaluate IV CDC administration; 3) the first double-blind, randomized, placebo-controlled trial of any form of cell therapy for treatment of DMD; and 4) the first clinical trial to yield evidence of therapeutic benefit in nonambulatory DMD patients. Two features of HOPE-2 are notable, teaching us new tricks that may be game-changing for future trials: 1) the move away from invasive cardiac-targeted cell delivery toward easily administered IV cell delivery; and 2) the use of sequential repeated cell doses. These innovations were driven by ever-increasing understanding of mechanism of action based upon preclinical studies.

### TISSUE ENGINEERED PATCHES OF PLURIPOTENT STEM CELL–DERIVED CMs FOR CARDIAC REPAIR.

The recognition that adult cells, even when collected from heart tissue, failed to generate CMs has fueled an intense research on human pluripotent stem cells (hPSCs), which include both embryonic stem cells (ESCs) and induced pluripotent stem cells (iPSC) and share the properties of indefinite self-renewal and pluripotency (12,13). Thus, hPSCs provide an unlimited source of relevant cell lineages to be used for cardiac repair. From a clinical perspective, pluripotent stem cell (PSC)–based cardiac derivatives are actually the only cells that can be scaled-up to generate the huge numbers of CMs required for effecting a true remuscularization, and although deriving CMs in sufficient numbers and with adequate quality for therapeutic applications used to be difficult (14), these challenges have now been largely resolved with improved directed differentiation protocols (15) and bioreactor cultures (16–18). For post-myocardial infarction (MI) cardiac repair, studies in rodent (survival demonstrated for up to 220 days) (16), guinea pig (survival demonstrated for 35 days) (19), porcine (survival demonstrated for 28 days) (20), and nonhuman primate (survival demonstrated for 90 days) (21) models have yielded compelling evidence in support of medium-term remuscularization. Importantly, although translational studies in large animals have actually demonstrated that macroscopic grafts of hPSC-derived CMs coupled to the native myocardium could be achieved with direct intramyocardial delivery of large cell doses (~10^9^ cells/animal), this benefit was obtained at the cost of a high incidence of ventricular arrhythmias, notably contributed by the usually immature phenotype of the PSC-differentiated CMs and the subsequent occurrence of an electrical mismatch at the graft/host interface. Another important finding of these preclinical studies has been that CM deposition and retention were enhanced when the cells are delivered via a tissue-engineering approach.

These 2 concepts, ie, the possibility to remuscularize the heart by PSC-derived cardiac cells and the efficacy of adjunctive biomaterials, have paved the way to the first clinical trials entailing the open-chest epicardial delivery of cell-laden patches in patients with advanced HF. One of them has used ESC-derived CPC embedded in a fibrin patch (ESCORT [Transplantation of Human Embryonic Stem Cell-derived Progenitors in Severe Heart Failure] Trial) (NCT02057900) (22) with a concomitant coronary artery bypass grafting and has successfully met its primary safety end point (the first patient who was operated on will reach a 7-year follow-up in October 2021). The other 2 trials, in which cell therapy is used as a stand-alone procedure, are ongoing and aim to deliver iPSC-derived CM under the form of a cell sheet (jRCT2053190081) (23) or a collagen-based construct (24) (BioVAT-HF [Safety and Efficacy of Induced Pluripotent Stem Cell-derived Engineered Human Myocardium as Biological Ventricular Assist Tissue in Terminal Heart Failure] Trial; NCT04396899). The latter trial is comprised of a dose-finding cohort (Part A; up to 18 patients to be treated) and an extension cohort (Part B, up to 35 patients to be treated) and is designed both to confirm safety for a broad range of engineered human myocardium (EHM) doses and to provide the first proof-of-concept for EHM-induced heart remuscularization in humans. Extensive investigations of EHM allografting in preclinical rat (25), mouse (26), and Rhesus macaque (unpublished data, oral communication, W.-H. Zimmermann) models found the following: 1) no palpable safety concerns (particularly no arrhythmia or tumor formation); 2) evidence for efficacy via the anticipated mechanism of action (ie, remuscularization) with enhanced local and global heart function; and 3) proof of long-term CM retention. Given this, clinical phase I/II testing is an important next step to scrutinize whether and how patients with advanced HF can benefit from hiPSC-EHM implantation.

So far, none of the study procedures have been associated with adverse events. Due the limited patient number in these first-in-man studies, conclusions as to efficacy are not yet possible. Should such an efficacy been demonstrated, it will be critically important to try to decipher its mechanism: true sustained heart remuscularization by PSC-derived CMs electromechanically coupled with host CMs or if the latter is prevented by the thin fibrotic barrier that commonly isolates the patch from the underlying myocardium, paracrine mechanisms, such as the release of exosomes carrying a myocardium-salvaging cargo, or thickening of the heart wall, thereby providing passive mechanical support, improving myocardial bioenergetics, and reducing wall tension according to the Law of LaPlace. The clarification of these mechanisms of action is critical in view its practical implications. Namely, all of these PSC-derived cells are allogeneic, including iPSC (their use as autologous iPSC is not realistic for cardiac repair in large patient populations because of logistical and economic reasons). Consequently, immunosuppression is required for keeping cells alive. If one targets long-term remuscularization, this immunosuppression needs to be lifelong, which raises the issue of the long-term adverse effects of these drugs (even though their dosing can likely be reduced compared with what is used in solid organ transplant recipients). Conversely, if reliance is on paracrine mechanisms, immunosuppression can be transient because it is only intended to give cells enough time to release the factors underpinning their effects. Currently, the time window during which this release occurs is not known, and in the ESCORT and Cell Sheet trial, the choice has been made to stop immunosuppression after 1–3 months.

Advances in methods to differentiate other cell lineages present in the heart including different populations of endothelial cells, such as arterial endothelial cells (27), cardiac fibroblasts (28,29), and distinct populations of hPSC-derived CMs (eg, atrial, ventricular), open new possibilities for advancing therapeutic approaches (30). On the other hand, advances in HLA-matching, gene editing technologies, and induction of tolerance could allow the generation of hPSC lines that may evade immune rejection, although each of these strategies is still fraught with uncertain safety issues (31). A last area where advances are required is the enhancement of PSC-derived cells maturation before transplantation to minimize subsequent arrhythmogenic events related to a still too early stage of CM cell differentiation.

### DELIVERY ISSUES.

If PSC derivatives are used, only direct myocardial delivery of the cells, either incorporated in a patch, as described in the previous text, or injected through an endoventricular catheter, is realistic to match the dual objective of maximizing the number of remuscularization-targeted cells in the diseased area while minimizing systemic off-target effects. The situation is different with adult cells, which offer more flexibility because in addition to the epicardial and endomyocardial routes, actually known to result in poor engraftment rates, a systemic delivery can also be considered. Although, from the onset, it may look counterintuitive in view of bio-distribution patterns of systemically infused cells showing that they are primarily home to the lungs, spleen, and liver with very few, if any, reaching the heart, there has been some evidence for their clinical efficacy, in particular in a randomized trial testing umbilical cord-derived MSCs (32). One current hypothesis is that remotely sequestered cells could tip the phenotypes of immune cells to promote a reparative and anti-inflammatory pattern (33), and that the activated immune cells would then convey the protective signaling molecules to the heart. If additional clinical trials confirm this hypothesis, then one of the primary mechanisms of action for cells could involve the endocrine regulation of myocardial inflammation, which is a hallmark of HF (34). Compared with other routes, the intravenous one is attractive because of its simplicity, low cost, and more importantly, its lack of invasiveness, which allows repeated dosing, which seems to be critical for achieving sustained benefits (8).

## TRANSITIONING TOWARDS CELL-DERIVED PRODUCTS

With accumulated experience, it has become apparent that transplanted cells did not survive in substantial amounts beyond a few weeks because of multiple factors (eg, hypoxic environment, shear stress during needle-based injections, loss of attachments to an extracellular matrix, inflammation, and rejection in case of allogeneic cells). The parallel observation that a functional benefit could still be present despite the physical clearance of the grafted cells has then led to the hypothesis that cells were not acting as a replacement therapy but rather as boosters of endogenous repair pathways through the release of a wide array of biomolecules endowed with tissue-repair properties. Most of these biomolecules are enclosed in extracellular vesicles (EVs) (which include exosomes), nanosized particles rich in proteins, noncoding nucleic acids, and lipid rafts that they can shuttle into recipient cells, thereby modulating multiple signaling pathways. To date, these pathways particularly involve a mitigation of inflammation and fibrosis, a stimulation of angiogenesis, and an enhancement of cellular energetics, while the proliferation of native CMs looks more uncertain, which fully justifies attempts at reinducing this proliferation by targeting endogenous cell cycle regulators, as discussed further. The most compelling evidence for the role of EVs as mediators of the effects of cell therapy comes from their ability to recapitulate the protective properties of the cells that produce them across a wide variety of preclinical disease models, including cardiac injury (8,35,36). Interest in the therapeutic potential of EVs is illustrated by already 22 clinical trials of EV treatments for a variety of disease states were registered on the ClinicalTrials.gov web site as of February 2021. This interest in EVs is largely driven by some of their features that make them attractive for clinical use: a manufacturing process more akin to that of a drug (and EVs are regulated as biological medications rather than ATMPs); a better control over batch-to-batch consistency and dosing; the availability of quality and potency controls that can provide straightforward readouts, while the EV cargo can be mapped via a variety of “omics” analyses; and the possibility of cryopreservation with only a modest decline in bioactivity, which makes them a readily available, off-the-shelf product. EVs are also less likely than cells to be associated with a variety of potential complications, such as arrhythmia, tumor formation, and alloimmunization (depending on the cell type [37]).

On the other hand, there is also substantial evidence supporting the hypothesis that injected stem cells can promote cardiac repair through the release of biologically active molecules acting in a paracrine fashion on adjacent cells (38,39), and distinct from those enclosed in EVs discussed in the previous text. Biologically active molecules that are secreted from injected stem cells include growth factors (vascular endothelial growth factor, hepatocyte growth factor, insulin growth factor 1) (40,41) and secreted Wnt inhibitors such as Sfrp2 (42,43). These paracrine factors improve cardiac function by promoting CM protection, survival, and proliferation (44–49); suppression of pro-inflammatory processes (50,51); reduced fibrosis (41,42); neovascularization (52,53); as well as prevention of metabolic changes associated with cardiac injury (54,55). A model of sequential paracrine relay has thus been proposed whereby biologically active molecules produced by the injected stem cells induce resident cardiac cells to secrete their own paracrine factors, which subsequently promote regenerative activity in other resident cells. Evidence for this paracrine relay arose from observations that paracrine factors released from MSCs induce macrophages to secrete plasminogen activator inhibitor 1 and stromal-cell–derived factor 1, which in turn promote endothelial cell differentiation (56). Similarly, MSCs induce macrophages to secrete interleukin 1 receptor antagonist, which influences the behavior of T cells natural killer cells, B cells, and dendritic cells (57–60). Future work will be necessary to determine if paracrine relays involve CMs and endothelial cells. Similarly, novel genetic models will need to be developed to investigate the paracrine relay model in more detail for clinical applications.

Taken together, these data highlight the possible interest of using cell-derived products as therapeutics for cardiac repair. Nevertheless, before moving to early trials, several issues still need to be addressed that particularly pertain to the selection of parental cells, large-scale production, and the specificity of purification. The most effective parental cells tend to have phenotypes that resemble cells in the targeted tissue (61), but at an earlier stage of differentiation (62); thus, 1 planned clinical trial will be conducted with EVs collected from iPSC-derived cardiovascular progenitor cells. Production of the parental cells can be scaled up with technologies such as bioreactors, and the large volume of conditioned medium can then be purified and concentrated via tangential-flow filtration, which appears to be most suitable for compliance with good manufacturing practices standards and can be adjusted (through the cutoff molecular weight of the filtration membrane) to select the fraction of the secretome intended for use. From this standpoint, the previously mentioned observations suggest that aiming at a highly purified exosomal fraction might be less efficacious than collecting most of the cellular secretome.

Finally, the mode of delivery of cell-derived products is as important as for cells. Different strategies are possible. The EV-enriched secretome can be injected directly into the myocardium, which raises the risk of a rapid wash-out weakening its expected benefits. To lengthen the exposure of the myocardium to the therapeutics, the secretome can thus be incorporated in slow-release hydrogels, which has usually produced superior outcomes (63). Last, but not least, the intravenous route leads, like for cells, to the predominant trapping of the secretome in lungs, spleen, and liver, but nevertheless has shown functional efficacy, possibly through a similar mechanism, ie, the secretome-induced shift of the phenotype of endogenous immune cells toward an anti-inflammatory pattern with remote benefits on the heart. Clearly, a major advantage of this route is the possibility of repeated administrations, which have been shown to sustain EV-induced functional improvements (8). To further increase the targeting of EVs toward the heart, they can be decorated with myocardium-specific ligands or rather targeted toward specific immune cell subtypes that can engulf and ferry them to injury sites (64).

## TOWARDS THE ENDOGENOUS GENERATION OF CARDIOMYOCYTES

The data presented in the preceding sections clearly show the following: 1) adult cells are unable to generate new CMs; 2) PSC-derived cells can successfully achieve this objective but at the cost of still clinically relevant issues (eg, arrhythmias, immune response); and 3) EVs are unlikely to induce CM proliferation, at least to an extent accounting for their protective effects (65). These observations explain the potential interest in switching from an exogenous to an endogenous strategy whereby native CMs would be forced to divide again. Indeed, for many years, scientists have been fascinated by the ability of species like newt or zebrafish to regenerate their heart following an injury. This capacity also exists in the mammalian heart but only shortly after birth, and it has long been thought that its loss was then irreversible. A better understanding of the mechanisms governing the cell cycle now raises the possibility that it might not be the case. In parallel, advances in cell reprogramming have also led investigators to consider an alternate approach aimed at converting heart fibroblasts into CMs

### EXTENDING THE NEONATAL MAMMALIAN REGENERATION WINDOW IN LARGE MAMMALS.

The discovery that neonatal mouse hearts exhibit regenerative capacity following injury provided evidence that mammalian hearts can regenerate; however, the time window of regeneration was found to be limited to the first week of life (66). Subsequent studies demonstrated that neonatal regeneration after injury also occurs in the large animal porcine MI models (67–69). Like the mouse studies, the porcine regenerative response was found to be limited to a few days following birth. The loss of regenerative capacity correlates with the transition in cardiac growth following birth from hyperplasia of CMs to hypertrophy. The signaling pathways and reversibility of this transition are under active investigation.

**FIGURE 1:**
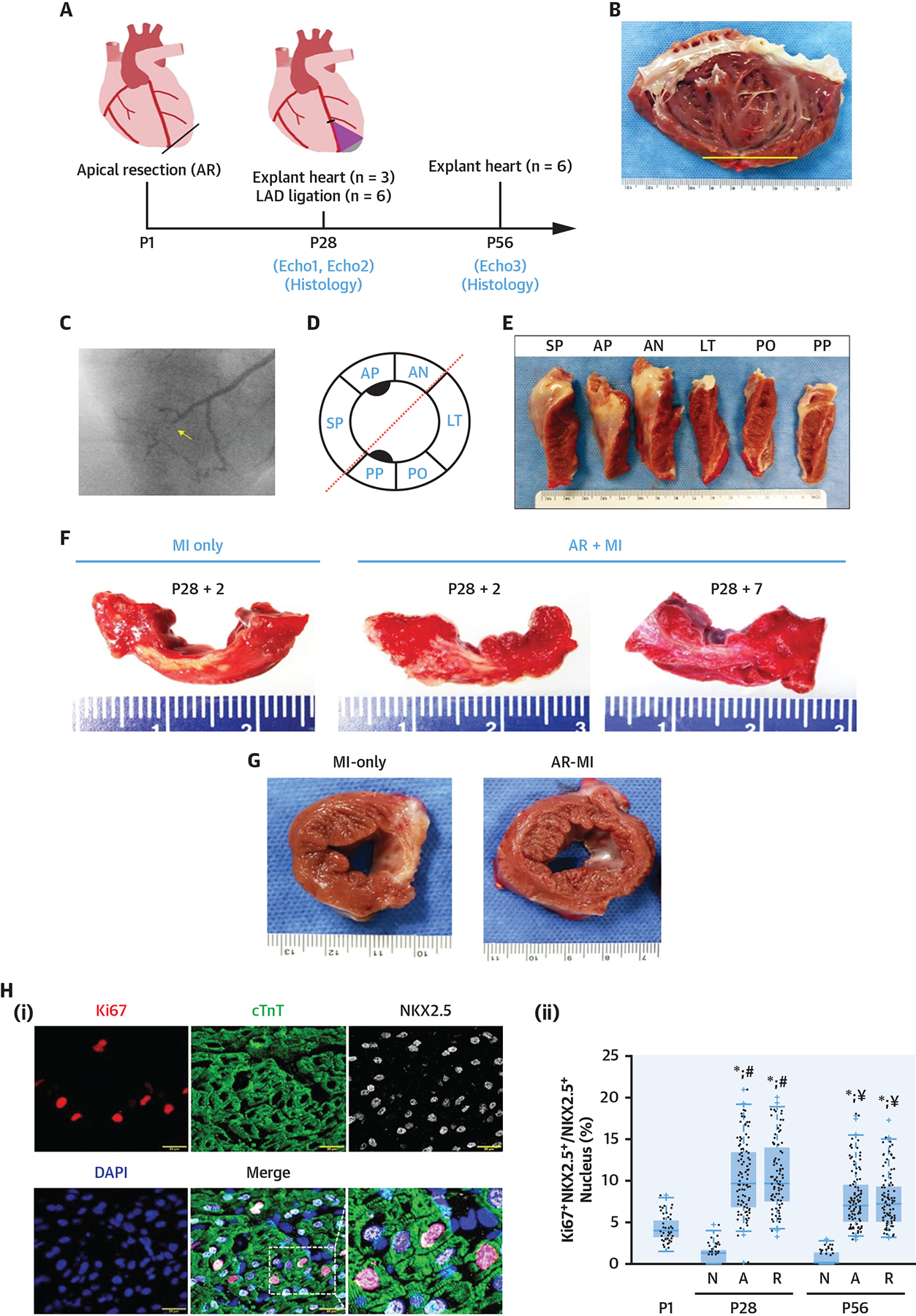
P1-Injury–Induced Activation of Cardiomyocyte Proliferation Prolongs Remuscularization of LV Infarct **(A)** Schematic of experimental protocol. **(B)** Typical inside-out view of left ventricular (LV) anterior wall of apical resection (AR)–myocardial infarction (MI) at P56. **(C)** Coronary angiographic image of AR + MI heart on P56; left anterior descending (LAD) occlusion is identified with an **arrow**. **(D)** Diagram of LV sectioning. **(E)** LV of an AR + MI heart on P56 was sectioned as shown in **D**. **(F)** LV anterior circumferential sections from MI-only and AR + MI hearts 2 (+2) and 7 (+7) days after MI induction. **(G)** Short-axis sections of MI-only and AR + MI hearts on P56. Scarred regions appear **white**. **(H)** Proliferating CMs were **(i)** identified via immunofluorescent staining for Ki67, cardiac troponin T (cTnT), and Nkx2.5, and then **(ii)** quantified on P1 (before AR surgery), and on P28 and P56 in normal hearts (N) and in the resected (A) and remote (R) regions of AR + MI hearts. AN = anterior wall; AP = left ventricular anterior wall with papillary muscle; DAPI = 4’,6’-diamidino-2-phenylindole; LT = left ventricular lateral wall; PO = left ventricular posterior wall; PP = left ventricular posterior wall with papillary muscle; SP = left ventricular septal wall.

To determine whether the proliferation machinery that was activated in CMs by myocardial injury on P1 could extend the window for myocardial regeneration in large mammals (69), injury was initially induced via apical resection (AR) on P1; then, the animals were allowed to recover until P28, when a second injury of MI was induced via permanent occlusion of the left anterior descending (LAD) coronary artery (ie, the AR + MI group). Control assessments were conducted in age-matched animals that underwent MI induction without previous AR surgery (ie, the MI-only group). MI induction led to a substantial myocardial scar that was observable 2–7 days after LAD artery ligation in both groups, and scarring persisted in the hearts of MI-only animals on P56; however, although the LAD artery occlusion remained visible in coronary angiography images of AR+MI animals on P56, there was no evidence of infarction in histological sections (Figure 1). Furthermore, when the number of CMs was calculated in LVs from AR + MI animals and from age-matched animals that did not undergo either surgical procedure (ie, naïve animals), CM numbers were significantly higher (by 40% on P28 and 92% on P56) in AR + MI hearts. This time-dependent increase in total CM number has also been observed in mice exposed to progressive hypoxia (70), and suggests that the remuscularization observed in AR + MI hearts was driven by the proliferation of endogenous CMs Markers for proliferation (Ki67, phosphorylated histone 3, and Aurora B) (71) also declined to nearly undetectable levels from P1-P28 in naïve hearts but increased in CMs of AR + MI hearts over the same period (Figure 1), and the frequency of proliferation marker expression did not differ significantly in CMs from the apically resected and uninjured myocardial regions, which suggests that AR surgery activated the proliferation machinery in CMs located throughout the entire LV, not just at the resection site. The results from single-cell and single-nucleus RNA sequencing (scRNA-seq and snRNA-seq, respectively) analyses also suggested that the initial injury extended CM proliferation window to P28, because AR surgery on P1 was associated with less mature patterns of CM gene expression, and bulk RNA sequencing of gene expression in the hearts of pigs that underwent MI or sham surgery on P1 identified 5 signaling pathways (mitogen associated protein kinase, HIPPO-YAP, cyclic AMP, Janus kinase/signal transducers and activators of transcription, and Ras) that were up-regulated in cardiac tissues collected from the MI group on P7 and/or P28 (72). Thus, although the time window for complete remuscularization of infarcted myocardium in neonatal large animals (pigs) normally closes within 1–2 days after birth, these results demonstrate that it can be extended until at least P28 by inducing myocardial injury (AR) on P1.

These findings are remarkably significant, because they are the first to demonstrate that CMs in the hearts of large mammals can be induced to proliferate and regenerate the contractile tissue that is lost to MI, and that the neonatal window for cardiac remuscularization can be extended up to P28 by inducing AR in P1 newborn large mammals (67,69,73). If this CM cell-cycle activation can be activated in neonates, the same signaling pathways may be activated in adults as well, which is highly impactful and significant. Planned investigations will expand upon the results from these studies with neonatal pigs: 1) by conducting scRNA-seq, snRNA-seq, and other state-of-the-art analyses to continue identifying/validating key regulatory pathways that govern the P1-injury–induced proliferation of neonatal CMs and drive the complete structural recovery of infarcted pig hearts; and 2) by “turning back the clock” in adult heart by using modified RNA or adeno-associated virus 9 to target the expression of CM cell-cycle regulatory molecules and promote remuscularization of adult large mammal hearts after MI. The challenges associated with exogenous cell transplantation, even if optimized by the combination with a tissue construct (see previous text), make this concept of rather leveraging the self-repairing capacity of the neonatal heart to increase the endogenous contractile cell pool strongly appealing.

In line with the previous findings, a recent study has used a pig model of ischemia-reperfusion to assess the effects of directly delivering into the myocardium an adenovirus-based siRNA aimed at inhibiting *Salv*, a component of the Hippo pathway that contributes to the nuclear exclusion of transcription factors driving pro-proliferative genes (74). This study reports an improved recovery of function in treated pigs with evidence for CM division. Taken together, these data open new attractive therapeutic possibilities with the caveat that the claims of CM proliferation need to be cautiously analyzed because the direct documentation of this event is still made technically challenging by the failure of commonly used cell cycle markers to unambiguously distinguish between polyploidy, endoreplication (DNA duplication without the completion of mitosis or cytokinesis), endomitosis (cell cycle passing with or without nuclear division but without cytokinesis), nuclear division (karyokinesis), and true cell division (cytokinesis) (75).

## DIRECT REPROGRAMMING OF CFs INTO CMs

Recent attempts to reprogram fibroblasts directly into CMs suggest that resident CFs associated with cardiac repair after injury or within the scar could be viable targets for remuscularization therapy.

A combination of 4 microRNAs (miR-1, -133, -208, and -499, collectively referred to as “miR combo”) successfully induced a CM-like phenotype in fibroblasts both in vitro and in vivo, and the same combination of factors was associated with modest, delayed, functional improvements in a murine model of myocardial injury (76). Subsequently, a series of mechanistic studies was performed to identify additional factors that could potentially be targeted to enhance the benefit of miR combo reprogramming, including epigenetic modifications, which strongly impact cell-fate specification during embryonic development. miR-combo–induced CF-to-CM trans-differentiation occurs, at least in part, via down-regulation of the trimethylated state of lysine 27 in histone 3 (H3K27me3). H3K27 is trimethylated by enhancer of zeste homology 2 (EZH2), and miR combo targets EZH2 for degradation (77); thus, miR combo indirectly reduces the occupancy of H3K27me3 on genes of the cardiac transcription factors, such as myocyte enhancer factor 2C, Gata4, and T-box transcription factor 5, which are required but not sufficient (78) for cardiac reprogramming. miR combo also functions as a ligand of Toll-like receptor (TLR) 3, one of a family of RNA-sensing receptors that also includes retinoic acid-inducible gene I (Rig-1), melanoma differentiation-associated protein 5 (Mda-5), TLR7, TLR8, and TLR9 (79). Upon binding to their specific ligands, RNA-sensing receptors activate a common signaling pathway that includes the transcription factors activator protein 1, interferon regulatory factors 3/7, and nuclear factor kappa B (NFkB) (79), which is consistent with observations that pharmacological TLR3 activators strongly enhance, while inhibitors of the TLR3-NFkB pathway block, the maturity of miR-combo–reprogrammed CMs (80). Synthetic RNA agonists of Rig-1 also enhanced the yield of mature CMs in response to treatment with miR-combo (81). Interestingly, miR-133 enhanced MEF2C, GATA4, and TBX5-induced direct cardiac reprogramming in human fibroblasts via targeting immune-related genes (82), suggesting distinct mechanisms underlying mouse and human reprogramming.

### HEART REGENERATION: BEYOND THE MERE REPLENISHMENT OF THE CM POOL.

Inducing the proliferation of CMs without providing them with the necessary nutritive supply required for their survival is unlikely to be functionally beneficial. Thus, cardiac regeneration should likely include a vascular component as a robust vascular network promotes oxygen delivery to the injured and border regions of the myocardium and thereby limits cardiac remodeling and progression to HF.

Therefore, strategies for enhancing cardiac vasculogenesis and cultivating a regenerative milieu are being pursued to promote remuscularization in the injured heart. Previous studies in multiple laboratories have shown that Ets Variant Transcription Factor 2 (*Etv2*), a master regulator of the endothelial lineage, can be manipulated to reprogram somatic cells into endothelial cells (ECs) (83). Master regulators also promote cell proliferation and cell migration, and scRNA-seq analyses demonstrated that Etv2 overexpression in endothelial progenitor cells increased the expression of cell-cycle regulatory genes, as well as *Yes1*, in a dose-dependent fashion. Furthermore, results obtained with bioinformatics analyses and molecular biology techniques suggest that Etv2 binds to the *Yes1* promoter and functions as a direct upstream regulator of *Yes1*, which subsequently activates Yes-associated protein (YAP), a downstream effector of the Hippo signaling pathway, to promote cellular proliferation, while scratch and sprouting assays indicated that the overexpression of Etv2 increased cell migration (84). Etv2 also functions as an upstream regulator of Ras homolog family member J (*Rhoj*) in endothelial progenitors, and the results from electrophoresis-mobility-shift and transcriptional assays, as well as mutagenesis studies and experiments with *Rhoj* shRNA, defined an important role for Etv2-Rhoj signaling in endothelial-lineage cells (85). Collectively, these recent studies establish the key role of Etv2-mediated networks in the functional regulation of endothelial progenitor cells and suggest that these networks could be targeted therapeutically to promote vasculogenesis and cardiac repair.

## CONCLUSIONS

After 2 decades and more than 200 patient studies (86,87), the results from mechanistic preclinical studies, continue to provide insights that can guide the clinical translation of cell-based therapies. From a translational perspective, new approaches to cardiac repair can be stratified according to their mechanistic targets and time frame. In the short-term, 2 main approaches can be considered (Central Illustration). The first entails the use of cells, primarily cardiac committed cells such as cardiospheres or PSC-derived CMs, with or without a shielding biomaterial, with the objective of repopulating the heart with new muscle cells that could engraft and improve its contractile function. The second approach rather aims at leveraging the paracrine effects of cells and switches from direct cell transplantation to the administration of their secreted products, including but not limited to EVs with the purported advantage of an easier implementation. The underlying premise, though, is no longer to generate new CMs but rather to modulate the innate immune system, shifting it toward limitation of inflammatory tissue damage. Whether reliance on exclusive cell products can improve clinical outcomes to the same extent as transplantation of the parental cells remains to be established.

In the longer-term, the recognition that the molecular and cellular basis for progressive HF is the result of the inability of damaged and apoptotic CMs to be replaced justifies to investigate approaches targeted at a more *direct* remuscularization of the injured LV by “turning back the clock” of CM cell-cycle or generating new CMs from other cell types such as fibroblasts. However, the efficiency and safety of these strategies, particularly their ability to generate CMs, seamlessly coupled with their native counterparts and to allow a regulation of these induced proliferative events preventing an uncontrolled and harmful cardiac growth, still need to be appropriately addressed before moving to clinical applications.

From the molecular and cellular perspective, HF occurs due to the loss of the contractile unit of the left ventricle: CM. In this paper, we have provided updates on CM regenerative potential in the neonatal injured heart of large mammal, the preclinical cell-based studies for the treatment of HF, as well as recent and proposed clinical trials of cell- or EV-based therapy. The implications of the results and observations presented here, as well as in the many excellent and exhaustive reviews of cardiac regeneration and repair that are available in the literature, continue to be vigorously debated. We believe the field benefits from such active discussion, which significantly advances the science. Going forward, from a translational perspective, we believe some key mechanistic questions deserve attention:

1. Can we identify and manipulate factors/signaling pathways that regulate the CM cell cycle, and “turn-back the clock” of CM cell-cycle to remuscularize hearts that have experienced MI?
2. If the option is to transplant cells, is it required that the cells feature a cardiac phenotype or cardiogenic potential? Also, should allogeneic progenitor cells be chosen, what is the best strategy to mitigate the expected immune responses with regard to the risk-to-benefit ratio?
3. If secreted factors from the transplanted cells are the basis of the benefit, and positive modulation of the inflammatory/immune response to cardiac injury is required, might intravenous cell delivery be preferable from a practical perspective?
4. In the future, should “exogenous” transplantation approaches (regardless of whether cells or cell products are used) be replaced by “endogenous” strategies aimed at stimulating innate mechanisms of CM proliferation and cross-talk among CM, vascular cells and the extracellular matrix?

From a clinical perspective, well-designed phase III trials are needed to determine whether cell or cell-product-based therapies are beneficial in patients with HF. We are hopeful that the ongoing and future preclinical and clinical studies will provide answers to these questions and contribute to better define the place that cell therapy at large might take within the armamentarium of HF treatments.

## Figures and Tables

**CENTRAL ILLUSTRATION:**
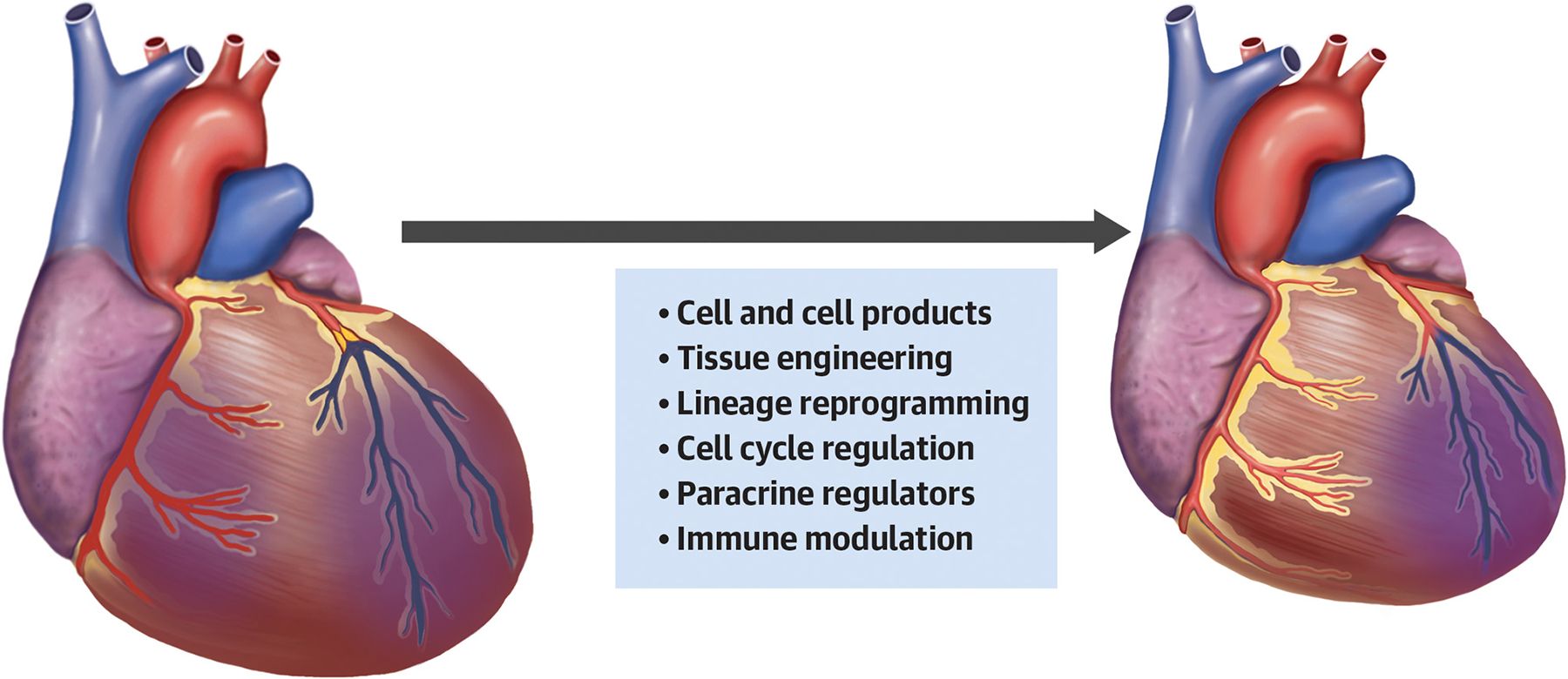
Cell and Cell Products for Cardiac Repair Postinfarction left ventricular remodeling–associated heart failure results in considerable morbidity and mortality. Current and future treatment strategies will likely involve repeated interventions using cell and cell products such as reprogramming strategies, engineered muscle constructs, and modulation of the cell cycle regulation or immune system responses.

## HIGHLIGHTS

- Cardiac cell therapy has been extensively studied, but mechanisms of action and clinical benefits have not been established.
- Cell-derived biomolecules and adjunctive biomaterials are key assets that may potentiate the efficacy of cell therapy.
- Strategies that promote CM proliferation and immunomodulation following cardiac injury hold promise for sustainable heart repair.

## ABBREVIATIONS AND ACRONYMS

AR: apical resection
CDC: cardiosphere-derived cell
CM: cardiomyocyte
CPC: cardiac c-kit positive progenitor cell
EV: extracellular vesicles (include exosomes)
HF: heart failure
MI: myocardial infarction
MSC: mesenchymal stromal cell
P1: postnatal day 1
